# An Adaptive Multi-D-Norm-Driven Sparse Unfolding Deconvolutional Network for Bearing Fault Diagnosis

**DOI:** 10.3390/s24082624

**Published:** 2024-04-19

**Authors:** Jianbo Lin, Han Zhang, Yunfei Li, Zhaohui Du

**Affiliations:** 1 School of Construction Machinery, Chang’an University, Xi’an 710064, China; 2021125081@chd.edu.cn (J.L.); 2022125066@chd.edu.cn (Y.L.); 2 Key Laboratory of Road Construction Technology and Equipment of MOE, Chang’an University, Xi’an 710064, China; 3 School of Marine Science and Technology, Northwestern Polytechnical University, Xi’an 710060, China; duzh@nwpu.edu.cn

**Keywords:** blind deconvolution, sparse optimization, adaptive period estimation, algorithm unfolding network

## Abstract

Impulsive blind deconvolution (IBD) is a popular method to recover impulsive sources for bearing fault diagnosis. Its underpinnings are in the design of objective functions based on prior knowledge of impulsive sources and a transfer function to describe transmission path influences. However, popular objective functions cannot retain waveform impulsiveness and periodicity cyclostationarity simultaneously, and the single convolution operation of IBD methods is insufficient to describe transmission paths composed of multiple linear and nonlinear units. Inspired by the MaxPooling period modulation intensity (MPMI) and convolutional sparse learning (CSL), an adaptive multi-D-norm-driven sparse unfolding deconvolution network (AMD-SUDN) is proposed in this paper. The core strategy is that one target vector with simultaneous impulsiveness and cyclostationarity is constructed automatically through the MPMI; then, this vector is substituted into the multi D-norm to design objective functions. Moreover, an iterative soft threshold algorithm (ISTA) for the CSL model is derived, and its iterative steps are unfolded into one deconvolution network. The algorithm’s performance and the hyperparameter configuration are investigated by a set of numerical simulations. Finally, the proposed AMD-SUDN is applied to detect the impulsive features of bearing faults. All comparative results verify that the proposed AMD-SUDN achieves a better deconvolution accuracy than state-of-the-art IBD methods.

## 1. Introduction

It is a basic task to extract impulsive sources from noisy observation signals in the field of fault diagnosis for rolling bearing health monitoring [1,2,3,4]. Where there are localized faults on the elements of rolling bearings, impulses are generated sequentially and periodic structures are also embedded into the impulses due to rotational movement [5,6]. After that, periodic transient impulses propagate along the complex transfer function of the transmission path to the position of the vibration sensors; observation signals are captured by sampling equipment. Moreover, due to interference signals from other mechanical components and the electromagnetic noise from sensors, a low signal-to-noise ratio (SNR) is unavoidable. Consequently, observation signals y(t) are often modeled as

(1) y(t)=f(t)∗x(t)+e(t)

where f(t) describes the influence of the transmission path, x(t) is the periodic impulse and e(t) denotes the noisy signal. Impulsive blind deconvolution (IBD) [7] aims to mitigate f(t) and e(t) to recover the focused feature x(t) from the observation signals y(t) of sensors. However, it is an ill-posed problem and there is no unique solution for x(t). Thus, prior knowledge of x(t) is exploited to solve this problem, and, naturally, different prior knowledge predicates various families of IBD techniques.

Firstly, the kurtosis property of impulsive sources x(t) is utilized to design IBD methods. The core idea behind kurtosis-based methods is to search one inverse filter $f^{-1}\left(t\right)$ to cancel the modulation function f(t) by maximizing the statistical indexes (such as kurtosis, Gary’s variable norm, skewness, sparsity, Gnini index); typical methods include MED, GMED, G-$l_{p}/l_{q}$ and Box–Cox sparse measures [8,9,10,11,12,13,14]. However, the kurtosis index is very sensitive to discrete impulses, and thus these methods often obtain a single impulse, which cannot achieve a satisfying deconvolution accuracy. To solve this problem, periodic information of x(t) is further exploited to enhance the kurtosis objective function. One representative method is maximum correlated kurtosis deconvolution (MCKD), and its key strategy is to fuse the periodic information into the kurtosis index [15]. The deconvolution results demonstrate the significant periodic structure of impulsive sources x(t). Furthermore, another method, multipoint optimal minimum entropy deconvolution (MOMEDA) [16], directly designs a target vector based on periodic information and the Dirac function to describe the feature signals x(t), and its deconvolution results nearly achieve the ideal accuracy. However, the deconvolution accuracy of the MCKD and MOMEDA methods is decided by the strict periodic information. In practical engineering applications, it is difficult to calculate or extract periodic information from noisy observation signals due to low-SNR conditions. Therefore, some stage-wise periodic detection methods have been developed and their main aim is to find the maximum peaks of the auto-correlation function of envelope signals [17,18,19,20].

On the other hand, due to the slip phenomenon of rolling bearings and the speed fluctuations of rotational machinery, assuming that fault impulsive signals are strictly periodic is unreasonable, and cyclostationarity knowledge is adopted to design an objective function. Excellent examples of this include cyclostationarity maximization blind deconvolution (CYCBD) [21] and its enhanced versions [22,23,24]. Based on the fact that sparse feature information in the squared envelope spectrum is equal to the cyclostationarity characteristic of x(t) [25], several sparse indexes have been introduced as an objective function in the squared envelope spectrum, including $l_{2}/l_{2}$, the Hoyer index, spectral negentropy [26], the generalized $l_{p}/l_{q}$ norm [27], squared-enveloped multipoint kurtosis [28], the Gini index [29,30], the spectral harmonics-to-noise ratio [31], and composite envelope negentropy [32]. However, feature waveforms with the maximum cyclostationarity index often demonstrate a periodic structure but do not necessarily have an impulsive pattern. Therefore, it is important to design an objective function which describes the periodic structure and impulsive waveform simultaneously.

Different from the inverse filter $f^{-1}\left(t\right)$ learning-based IBD methods above, the convolutional sparse learning (CSL) strategy directly models the influence of the transmission path as a convolutional operation f(t)∗x(t), and its optimized filter f(t) reflects the unit impulse response of the transmission path [33]. Later, negative bounded prior knowledge was introduced into the CSL model to extract the envelope waveform of impulsive features, significantly improving the deconvolution accuracy [34]. Moreover, the low-rank regularized denoiser and the CSL deconvolution model are combined to extend the CSL model to low-SNR conditions [35]. A noise-aware statistical threshold is designed to automatically remove noise and achieve superior performances in IBD tasks [7]. Some other CSL-based IBD methods adopt the shift-invariant dictionary [36,37], local matching pursuit [38,39], and sparse filters [40] to detect impulsive sources. The nonlocal similarity of feature waveforms is also exploited to further enhance the deconvolution performance of the CSL model [41]. The core assumption of these CSL strategies is that transmission path influences can be approximately described by convolution operations, which are linear. However, for mechanical systems composed of many components, the transmission path includes linear and nonlinear units due to the diverse characteristics of the mechanical component materials, and thus it is inappropriate to utilize only one convolution for complicated mechanical systems. Consequently, deep neural networks are introduced to describe the nonlinear modulation relationships of transmission paths. A single linear layer combined with a backward automatic differentiation strategy is designed for IBD tasks [42]. A single convolution layer is also developed via the L-BFGS algorithm to recover impulsive signals [43,44]; furthermore, multiple convolution layers are optimized based on the maximum correlated kurtosis via a layer-wise search strategy [45]. These deep-network-based IBD methods mainly utilize a single linear or convolutional layer to design the mapping function, and there is also a lack of nonlinear activation units; therefore, these deconvolution networks have no capability to describe transmission path influences composed of linear and nonlinear units. It is thus another challenging problem to design one deep network with multiple linear and nonlinear units for a higher deconvolution accuracy.

To address the two issues above, an adaptive multi-D-norm-driven sparse unfolding deconvolutional network (AMD-SUDN) is proposed in this paper for bearing fault diagnosis. Firstly, one target vector with simultaneous impulsiveness and cyclostationarity is constructed automatically through the MaxPooling period modulation intensity (MPMI) method, and then this vector is substituted into the multi D-norm to design the objective function of the AMD-SUDN model. Then, based on the CSL deconvolution model and the algorithm unfolding framework [46], the iterative shrinkage threshold algorithm (ISTA) is adopted to solve the CSL model and then its iterative steps are unfolded into one SUDN with many linear and nonlinear activation units. The effectiveness of the SUDN in bearing fault diagnosis has been preliminarily verified [47]. Impulsive sources are extracted by the Adam optimizer using the PyTorch framework. Numerical simulations are then implemented to evaluate its deconvolution performance, and the superiority of the proposed AMD-SUDN method is investigated with respect to state-of-the-art IBD techniques. Lastly, one experimental study on CWRU rolling bearing data further validates that the AMD-SUDN achieves a better deconvolution accuracy.

The main contributions of this paper are summarized as follows.

1. MPMI and multi D-norm are combined to construct an objective function with simultaneous waveform impulsiveness and periodicity cyclostationarity, which to the best of our knowledge has not been investigated in the fault diagnosis field.
2. The iterative steps of the CSL model are unfolded into one deconvolution network, which is proposed to describe the linear and nonlinear units of the transmission path, which is a new way to utilize model-driven networks for IBD tasks.
3. Numerical simulations and deconvolution results demonstrate that the proposed AMD-SUDN outperforms the existing state-of-the-art IBD methods.

The rest of the paper is organized as follows. Section 2 will review the related works on the multi D-norm and fault period estimation. The CSL model and design process of the AMD-SUDN will be elaborated in Section 3. Numerical simulations are implemented to investigate the AMD-SUDN’s performance in Section 4, and experimental validations are demonstrated in Section 5. Finally, conclusions are drawn in Section 6.

## 2. Related Work

### 2.1. MOMED Blind Deconvolution Method

The MOMED method utilizes the multi D-norm of the filtered signal as an objective function and it assumes a deconvolution target with multiple impulses at known locations, which can be determined by the fault frequency of rolling bearings. The multi D-norm of the filtered signal can be expressed as follows:

(2) $$MDN\left(x\right)=\frac{1}{∥t∥}\frac{t^{T}x}{∥x∥}$$

where t is a target vector consisting of constants that determine the position and weight of the target pulse train to be recovered. The MOMED method confirms the inverse filter $f^{-1}$ by maximizing the MDN(x) objective function,

(3) $$\max_{f^{-1}}MDN\left(x\right)=\max_{f^{-1}}\frac{t^{T}x}{∥x∥},\;s.t.\;x=f^{-1}*y.$$

The local maximum value can be determined by taking the derivative with respective to the filter coefficient $f^{-1}$,

(4) $$\frac{d}{df^{-1}}\left(\frac{t^{T}x}{∥x∥}\right)=0$$

Then, we can obtain one closed-form solution

(5) $$\frac{t^{T}x}{{∥x∥}_{2}^{2}}f^{-1}={\left({\mathrm{YY}}^{T}\right)}^{-1}Yt$$

The impulsive source signal x can be calculated by the observation signal $y=[y_{1},y_{2},\cdots y_{N}]$ and the inverse filter $f^{-1}={\left[f_{1},f_{2},\cdots ,f_{N}\right]}^{T}$ as follows:

(6) $$x=f^{-1}*y$$

The MOMED method utilizes a target vector t to determine the position and weight of the periodic pulse components to be recovered, which is well suited for extracting impulsive features with special periodic patterns. However, in the fault feature detection of rolling bearings, strict periodic information is required to design a target vector t. Moreover, a target vector t with a strict periodic structure cannot reliably describe the slip phenomenon and rotational speed fluctuations, which seriously limits the efficiency of the MOMED method.

### 2.2. Period Modulation Intensity (PMI) Method

To detect the periodic information embedded into the observation signal y, the PMI method was developed, and its core operation is to find the maximum value of the auto-correlation (AC) function and then view the first maximum point as periodic information. Firstly, based on the fact that the envelope waveform obtained by the Hilbert demodulation transformation significantly enhances fault information compared to the original signals, it is feasible to utilize the envelope waveform to design an impulsive detection index. Then, the envelope waveform a(t) is divided into two parts: a periodic impulsive modulation component p(t) and an interference modulation component n(t). Moreover, p(t) and n(t) are linearly independent. The auto-correlation function of the envelope signal a(t) with time delay τ can be calculated as:

(7) $$Cor\left(a\left(t\right),a\left(t+\tau \right)\right)=\left\{\begin{array}{cc} E_{p}+E_{n}, & \tau =0\\ Cor(p(t),p(t+\tau )), & \tau \neq 0 \end{array}\right.$$

where $E_{p}$ and $E_{n}$ are the energies of p(t) and n(t), respectively. Let *T* be the time period of the impulsive component p(t); then, the AC function of p(t) is:

(8) $$Cor\left(p\left(t\right),p\left(t+iT\right)\right)=Cor\left(p\left(t\right),p\left(t\right)\right)=E_{p},\;i=1,2,\cdots$$

The PMI is thus a quantization index for impulsive information similar to signal-to-noise ratio (SNR). Finally, the PMI index can be modified to confirm the periodic information of impulsive features:

(9) $$PMI\left(\tau \right)=\frac{Cor(a(t),a(t+\tau ))}{Cor(a(t),a(t)-Cor(a(t),a(t+\tau )))}$$

The function PMI(·) only has local maximum values at τ=iT(I=1,2,⋯). Thus, these local maximum values and their relationships are utilized to confirm the first local maximum point (except τ=0), and its time lag τ is set as the impulsive periodicity *T*.

## 3. Proposed AMD-SUDN Deconvolution Method

**Figure 1:**
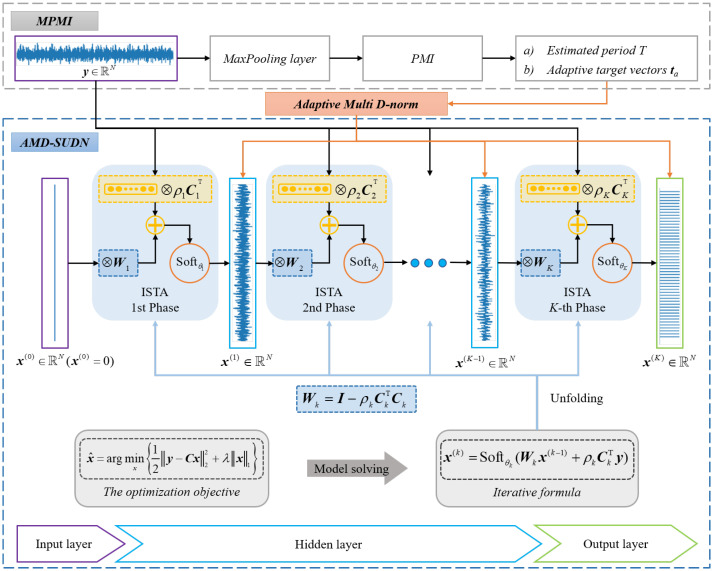
Flow diagram of the proposed AMD-SUDN.

Inspired by convolutional sparse learning and algorithm unfolding, a sparse deconvolution model is established and its ISTA solver is also derived; then, its iterative steps are unfolded into a sparse unfolding deconvolution network. On the other hand, to make the objective function retain waveform impulsiveness and periodicity cyclostationarity simultaneously, an adaptive multi D-norm is designed through the MaxPooling PMI method, which sidesteps the assumption that the target vector of the multi D-norm objective function depends on the strict periodicity of the fault waveform. The proposed AMD-SUDN structure is shown in Figure 1, and mainly consists of a convolutional sparse learning model, an ISTA solver and a sparse unfolding deconvolution network. These core modules in the AMD-SUDN will be described in the following subsections.

### 3.1. Convolutional Sparse Coding Model

In general, a rolling bearing fault signal y is a mixture of different components affected by complex transmission paths, which can be expressed as:

(10) y=f∗x+e

where x and e denote impulsive sequences and noise disturbances, respectively. f is the corresponding transfer functions of the path between impulsive sources and sensors. ∗ denotes the convolution operation. Due to the convolution operation ∗ being a type of linear mapping, the convolution model (10) can be rewritten in matrix–vector multiplication format,

(11) y=Cx+e

Deconvolution methods generally recover impulsive features by constructing an inverse filter, while convolutional sparse learning methods extract impulse features by directly modeling the modulation process of the transmission path, and thus the CSL method achieves a higher deconvolution accuracy.

Based on the fact that most points in the impulsive source x are zeros and thus x has a sparse pattern, the CSL method introduces a sparse regularization term to describe this sparse pattern; moreover, to guarantee the computational efficiency of the objective function, a sparse metric $l_{1}$ is adopted. The resulting optimization problem can be written as

(12) $$\hat{x}=\arg\min_{C,x}\left\{\frac{1}{2}{∥y-\mathrm{Cx}∥}_{2}^{2}+\lambda {∥x∥}_{1}\right\}$$

where λ is the equilibrium parameter. The CSL model is concise and easy to interpret; however, hyperparameter selection for C (filter length and initialization) and λ often requires expert knowledge. However, the optimization problem is nonconvex and there are many local minima. More importantly, there is only a single convolution operation to describe the transmission path’s influence with linear and nonlinear units, which is unreasonable for bearing fault diagnosis. Therefore, it is necessary to design a deep complex network with multiple linear layers and a nonlinear activation function for IBD tasks.

### 3.2. Sparse Unfolding Deconvolution Network

In this section, the convolutional sparse learning model is solved first using an ISTA solver while the filter variable C is fixed. The iterative steps are then unfolded into linear layers and soft-threshold activation is used to design a deep sparse network. Moreover, the filter variable C and impulsive features x are set as learnable parameters of the deep sparse network, which achieves one goal of obtaining one deep deconvolution network for transmission paths with linear and nonlinear units.

To solve problem (12), the ISTA algorithm perform gradient descent steps and proximal operator steps alternatively, where the iterative formulas can be written as,

(13) $$\begin{array}{cc} r^{\left(k\right)} & =x^{\left(k-1\right)}-\rho C^{T}\left({\mathrm{Cx}}^{\left(k-1\right)}-y\right)\\ \; & ={\mathrm{Wx}}^{\left(k-1\right)}+\rho C^{T}y \end{array}$$

(14) $$x^{k}={\mathrm{Soft}}_{\theta }\left(r^{k}\right)=\mathrm{sign}\left(r^{k}\right)\max\left(\left|r^{k}\right|-\theta \right)$$

where $C^{T}\left({\mathrm{Cx}}^{\left(k-1\right)}-y\right)$ denotes the gradient of the fidelity term ${∥y-\mathrm{Cx}∥}_{2}^{2}$, $r^{k}$ denotes the corresponding gradient descent term, ρ denotes the step size, *k* denotes the ISTA iteration index, W is used to replace $I-\rho C^{T}C$ for the learnable parameter, ${\mathrm{Soft}}_{\theta }\left(\cdot \right)$ denotes the soft threshold function, and θ denotes the threshold value.

The algorithm unfolding framework provides a way to design a network by unfolding the iterative process above into the architecture of the neural network, which is shown in Figure 1. Firstly, the gradient descent step is set as linear layers with learnable parameters C and ρ, and W is fixed as $I-\rho C^{T}C$ to reduce the network parameters. To improve the computation efficacy, $C_{k}$ and $C_{k}^{T}$ are a one-dimensional convolution operation and its transpose convolution operation, respectively. In order to ensure that the feature mapping length after each convolution is the same, a zero-padding operation is applied to feature mappings before the convolution operation; i.e., every feature mapping is filled with L/2 zero points, where *L* is the convolution kernel length.

Then, the proximal operator step or soft-threshold function is set as the activation function with learnable parameter θ. Moreover, to guarantee the excellent fitting capability of the sparse unfolding network, *K* iterative steps are expanded and thus there are *K* feature mappings, similar to $x^{\left(k\right)},k=1,\cdots ,K$, which will be constrained in the objective function to accelerate network training. As the expanded layers *K* increase, the network has more parameters and a better fitting capability, naturally leading to a better deconvolution performance, but the back-propagation procedure of gradient information is more complex and thus the developed network is more prone to a training trap. It is important to set the expanded layers *K* and convolutional kernel length *L* in the sparse network, and these parameters will be selected based on numerical analysis.

### 3.3. MaxPooling-Based Periodic Estimation

Although the envelope signal obtained by Hilbert demodulation usually has clearer fault information than the original signals, the envelope signal is often affected by the period of other high-frequency components and noises. P. Borghesani et al. [48] found that the nonlinear effect of the maximal pooling layer is quite different for high-frequency and low-frequency signal components. For any periodic component with a period of *T* time steps, if the pool size is chosen to be P≥T, the output features of the MaxPooling layer are almost identical to the envelope + down sampling operation, i.e., the envelope amplitude of the signal is extracted. On the contrary, if the pool size is smaller than the period of the signal (P<T), the overall trend in the original signal is preserved in the downsampled signal. Therefore, the MaxPooling layer provides a way to adaptively extract the envelope signals for a target vector t. Then, the MaxPooling period modulation intensity (MPMI) strategy and auto-correlation sequence are combined to identify impulse locations.

The MPMI strategy is implemented as:

(15) $$MPMI_{y}\left(\tau ,P\right)=\frac{Cor(MP_{y,P}\left(t\right),MP_{y,P}\left(t+\tau \right))}{Cor\left(MP_{y,P}\left(t\right),MP_{y,P}\left(t\right)\right)-Cor\left(MP_{y,P}\left(t\right),MP_{y,P}\left(t+\tau \right)\right)}$$

where $MP_{y,P}\left(t\right)$ denotes the maximum pooled envelope signal obtained by the maximum pooling layer and *P* denotes the pool size and step size of the maximum pooling layer. It is worth noting that the maximum pooling layer performs a downsampling operation on the measurement signal y, and one sample point after pooling denotes *P* sample points of the original signal, so the MPMI is plotted by multiplying its delay τ by the pool size *P* to ensure the matching of the signal dimensions.

**Figure 2:**
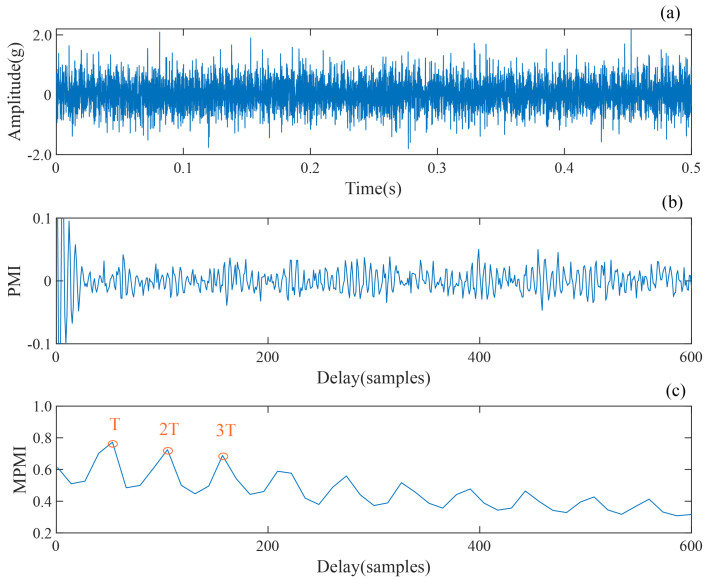
Comparative analysis of PMI and MPMI indexes: (**a**) time-domain waveform of the simulated signal, (**b**) PMI spectrum of the Hilbert envelope signal, and (**c**) PMI spectrum based on the MaxPooling operation.

To illustrate the superiority of the MPMI in identifying impulsive periodicity, one simulated signal, as well as the PMI of the envelope signal and the MPMI based on the MaxPooling layer, is given in Figure 2. The simulated signal is a mixture of amplitude-modulated periodic pulses and white noise with zero-mean, a variance of 0.4, an SNR of −4.47 dB, and a pulse period *T* of 57 sampling points. A series of weak periodic pulses can be observed in Figure 2a,b, showing that the period detection result of the PMI is affected by the high-frequency component, whereas the period detection result of the MPMI shown in Figure 2c is very significant, and T=56 sample points can be obtained by taking the interval mean of the extreme value as the estimated fault period.

### 3.4. Optimization Objective and Solver

The target vectors of the classic multi D-norm are strict periodic sequences based on a given impulsive period, which often is inconsistent with the actual fault sources because periodic pulses of rolling faults are not strictly periodic sequences due to phenomena such as slippage. In order to further remove the strong period constraints of the multi D-norm, an adaptive multi D-norm is designed by substituting the target vector t of the original multi D-norm with an adaptive target vector $t_{a}$ according to the MPMI. Specifically, the above MPMI index measures the correlation between the signal with time delay and the original signal. According to the correlation curves of the periodic signal, each extremely large value point in the MPMI index can be selected as an impulsive position to obtain one adaptive target vector $t_{a}$. The vector $t_{a}$ decides the impulse locations and periodicity from data point adaptively and thus it is consistent with the actual fault features, and this can remove the strong period constraint. The adaptive multi D-norm (AMD) can be written as:

(16) $$t_{a}=\delta \cdot \mathrm{Lmax}\left(MPMI_{y}\left(\tau ,P\right)\right)$$

where δ denotes the pulse function and Lmax denotes the set of auto-correlation maximum points, so $t_{a}$ has a pulse at each maximum of the signal.

The SUDN can be optimized by maximizing the AMD objective function. Firstly, the initialization of parameters and the setting of hyperparameters are needed, such as the pool size *P* of the MaxPooling layer, the unfolding number *k*, and the convolutional kernel length *L*. Secondly, the adaptive multi D-norm is obtained as the loss function of the sparse unfolding deconvolution network via the MPMI strategy, and the parameters of the whole AMD-SUDN (including $C_{k}$, $\rho_{k}$ and $\theta_{k}$) are optimized using the back-propagation algorithm for neural networks.

It is worth noting that each layer of the AMD-SUDN is equivalent to an iterative process of the ISTA, and the output of each layer of the network is equivalent to the result of one iteration, so the proposed adaptive multi D-norm can be constrained on the output of each layer of the network, which further enhances the performance of the unfolded network. The modified loss function of the AMD-SUDN can be formulated as:

(17) $$L\left(\Theta \right)=\sum_{k=1}^{K}AMD\left(x^{\left(k\right)},t_{a}\right)$$

## 4. Performance Analysis

In this section, the simulation signals of a rolling bearing fault are used to evaluate the effectiveness of the proposed AMD-SUDN. The vibration signals of a rolling bearing can be written as follows.

(18) $$y\left(t\right)=\sum_{i}A_{i}h_{i}\left(t-iT_{a}-v_{i}\right)+\sum_{j}B_{j}sin\left(2\pi f_{j}t+\varphi_{j}\right)+e\left(t\right)$$

where y(t) represents the fault vibration signal, which is synthesized from three components. The first one is the periodic pulse caused by the bearing fault, where $A_{i}$ and $T_{a}$ denote the amplitude and time interval of the pulse sequence, respectively, and parameter $v_{i}$ is used to simulate the random slip of the roller in the bearing, which is set to 1–2% of $T_{a}$. The second component is the harmonic disturbance, which is used to simulate the vibration generated by gear meshing or shaft rotation. $B_{j}$, $f_{j}$ and $\varphi_{j}$ indicate the amplitude, frequency and phase of the harmonic components, respectively. The last component e(t) represents the background noise. h(t) represents the impulse response function of the undamped single-degree-of-freedom system.

(19) $$h\left(t\right)=e^{-\xi t}sin\left(2\pi f_{d}t\right)$$

where ξ and $f_{d}$ denote the resonance damping coefficient and frequency, respectively.

**Figure 3:**
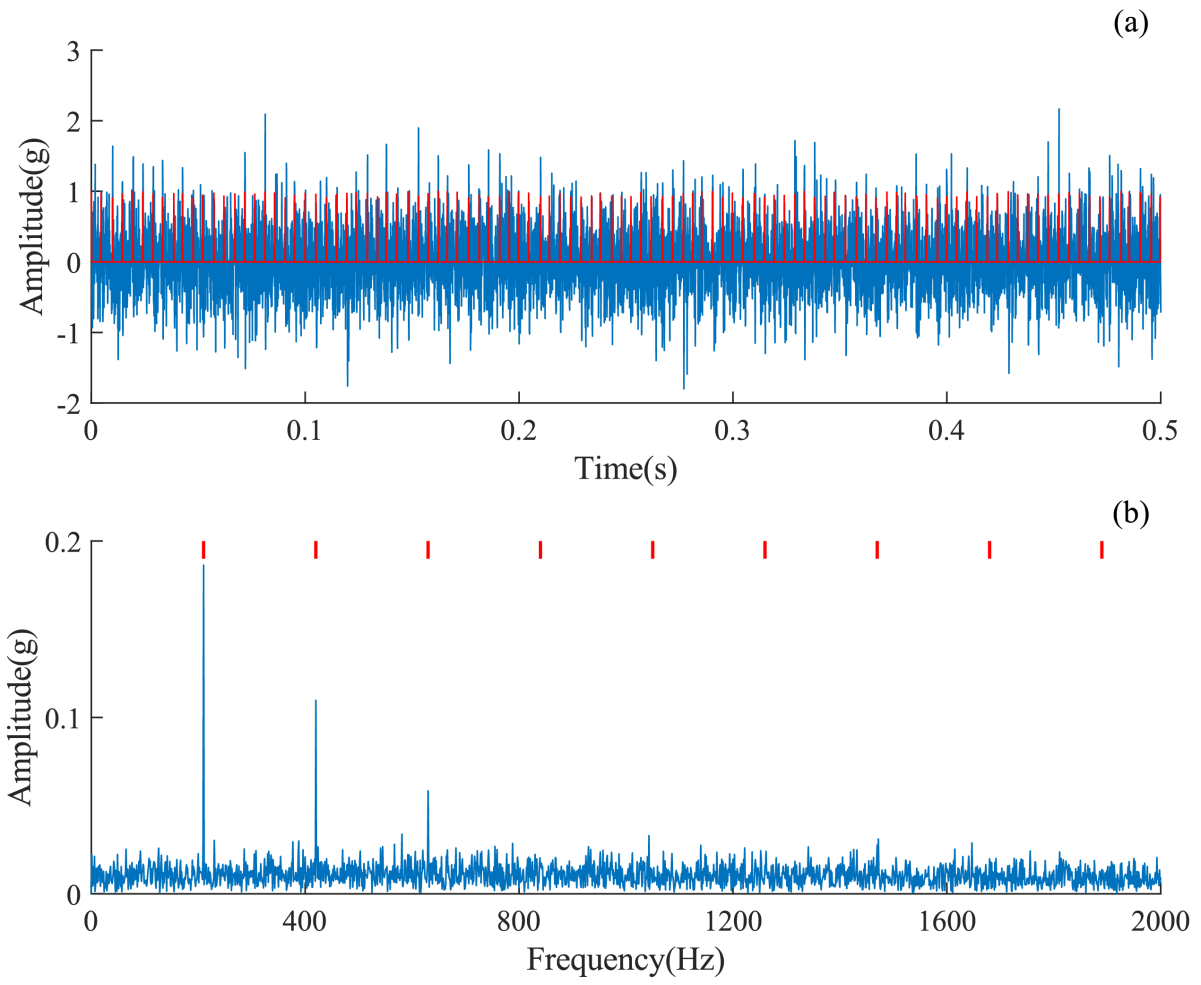
The simulation signal. (**a**) Waveform of the signal, (**b**) envelope spectrum of the signal.

The simulation signals of bearing outer ring faults have 6000 points with a duration of 1 s. Their detailed parameters are shown in Table 1, where $F_{s}$ denotes the sampling frequency, FCF denotes the fault characteristic frequency of the rolling bearing, and SNR is the signal-to-noise ratio. Figure 3a plots the time-domain waveform of the simulated signal, where the red curve indicates the outer ring fault feature, from which it can be seen that the outer ring fault feature is submerged by the noise. Figure 3b further displays the envelope spectrum. The first three orders of FCF are clearly visible.

### 4.1. Parameter Analysis of the AMD-SUDN Method

In the above Section 3.2, it can be seen that the hyperparameters of the AMD-SUDN have an important impact on its performance. The following parameters of the AMD-SUDN are selected by simulating signals: the filter length *L*, the network expansion depth *K*, and the pool size of the maximum pooling layer *P*. The experimental parameters are set as follows: epoch = 100, learning rate = 0.01, and optimizer = Adam.

Firstly, the hyperparameters *L* and *K* of the network are analyzed, and the multi D-norm and processing time are used as evaluation indexes. The experimental results are shown in Table 2. Classical MOMED requires longer filters to obtain better results. In order to choose the appropriate *L* value, the unfolding number *K* = 3 is fixed first, and then a series of values of *L* are selected for performance analysis. It is seen that the multi D-norm is higher as *L* increases, while the processing time of the network model is basically maintained at 1.8 s. According to these results, *L* is set to 400, and the effect of the unfolding number *K* is then evaluated. It can be seen from Table 2 that the network model performance first improves and then remains stable as *K* increases, but the processing time is proportional to *K*. Therefore, the expansion number *K* is set to 3.

**Figure 4:**
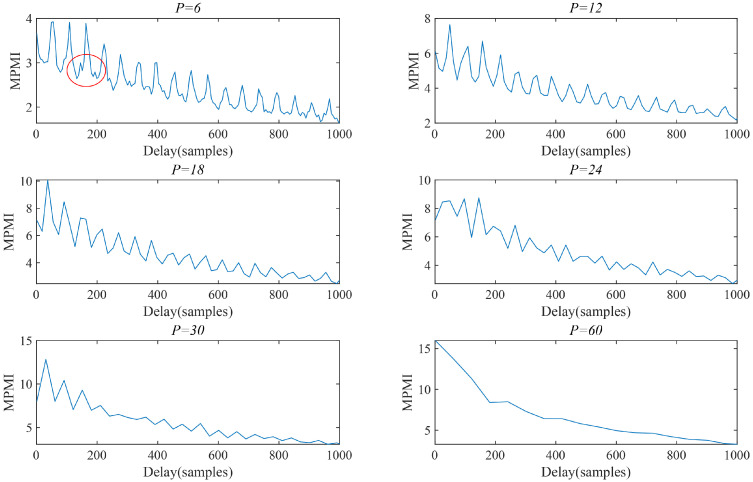
The effect of pool size *P* on MPMI.

Secondly, the effect of the pool size *P* on the AMD-SUDN needs to be further analyzed, because the envelope effect of MaxPooling can achieve good envelope demodulation only when *P* lies between the period of the high-frequency component and the period of the low-frequency fault features. Based on the simulated signals, it can be shown that the period of the high-frequency component is 6 sampling points and the period of the fault feature is 57 sampling points. Figure 4 displays the MPMI curves under different pool sizes *P*. It can be seen that when *P* is slightly larger than the period of the high-frequency component, the period of the fault feature can be confirmed more accurately, but when *P* = 6, a small amount of interference exists in the MPMI, and when *P* is close to the fault period, the MaxPooling envelope effect is reflected in the extraction of the amplitude, and the fault period is no longer extracted. Therefore, the pool size *P* is set to 12.

### 4.2. Interpretability Analysis of the AMD-SUDN

**Figure 5:**
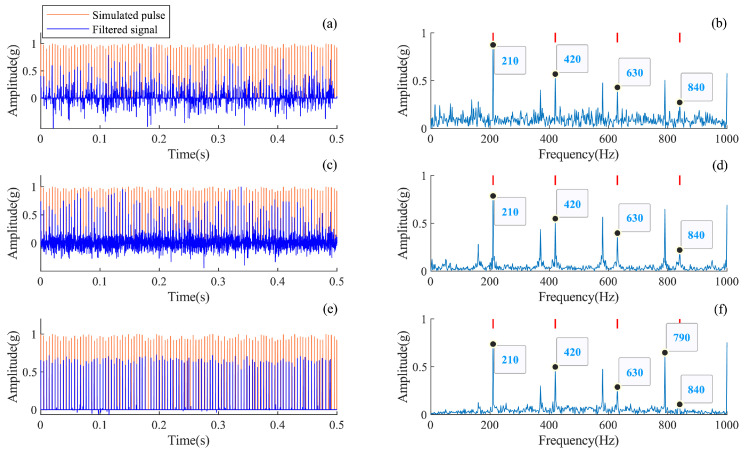
Filtered signal for each layer of the AMD-SUDN. (**a**) Waveform of the first layer, (**b**) envelope spectrum of the first layer, (**c**) waveform of the second layer, (**d**) envelope spectrum of the second layer, (**e**) waveform of the final layer, (**f**) envelope spectrum of the final layer.

Theoretically, the unfolding network inherits the interpretability of the iterative algorithm, and the interpretability of the AMD-SUDN is examined in this subsection. Based on the parameter selection above, the output waveform of each layer of the unfolding network and their envelope spectra are plotted in Figure 5. From these results in Figure 5a,c,e, it can be seen that the impulse features of the filtered signal become more significant as the network unfolds. From the envelope spectra in Figure 5b,d,f, it is found that the FCF and its harmonic components are more significant as the network unfolds, and the final extracted impulse sequence is very significant, as seen in Figure 5e,f. This further verifies the stage-wise interpretability of the unfolded network along the iterative steps.

It is worth noting that since the downsampling operation of the maximum pooling layer also reduces the frequency from 12 kHz to 1 kHz, the frequency range of the extracted fault pulse in the envelope spectrum is reflected back at 1 kHz, resulting in the phenomenon of mixing of some frequency bins, which can be observed in Figure 5f. The frequency component of 790 Hz, which is reflected back from 1 kHz, is spaced at a frequency interval of 210 Hz from the fault pulse frequency.

### 4.3. Comparison of Deconvolution Methods

The proposed AMD-SUDN method is compared with three deconvolution methods, MCKD, CYCBD, and BAD-MOMED, in this subsection. It can be seen that all three methods require pre-given theoretical fault periods. As determined through preliminary experiments, the number of iterations for all three deconvolution methods is set to 100, with the filter lengths of CYCBD and MCKD set to 40 and the number of MCKD shifts set to 1. The specific details of BAD-MOMED can be found in [42], in which all filter lengths are consistent with the proposed method, set to 400, and implemented in a three-filter cascade corresponding with AMD-SUND’s unfolding number *K*.

**Figure 6:**
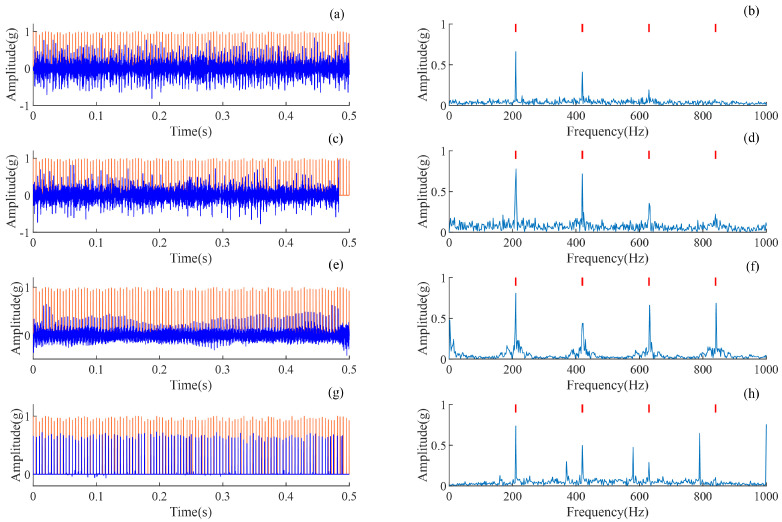
Deconvolution results of the simulated signal. (**a**) Filtered signal of MCKD and (**b**) its envelope spectrum, (**c**) filtered signal of CYCBD and (**d**) its envelope spectrum, (**e**) filtered signal of BAD-MOMED and (**f**) its envelope spectrum, and (**g**) filtered signal of the AMD-SUDN and (**h**) its envelope spectrum.

The deconvolution results of all comparison methods and their envelope spectra are shown in Figure 6. From the time-domain waveform in Figure 6a,c,e,g, it can be seen that the sparsity and periodicity of the shock force sequences extracted by the proposed method are more significant compared to other deconvolution methods. Nearly all noise is removed; however, comparative methods contain strong noise and the impulse waveform is unclear. From the envelope spectra of Figure 6b,d,f,h, it can be seen that all methods can detect FCF and higher orders. Therefore, the proposed AMD-SUND method shows a better deconvolution accuracy for rolling bearing fault diagnosis.

**Figure 7:**
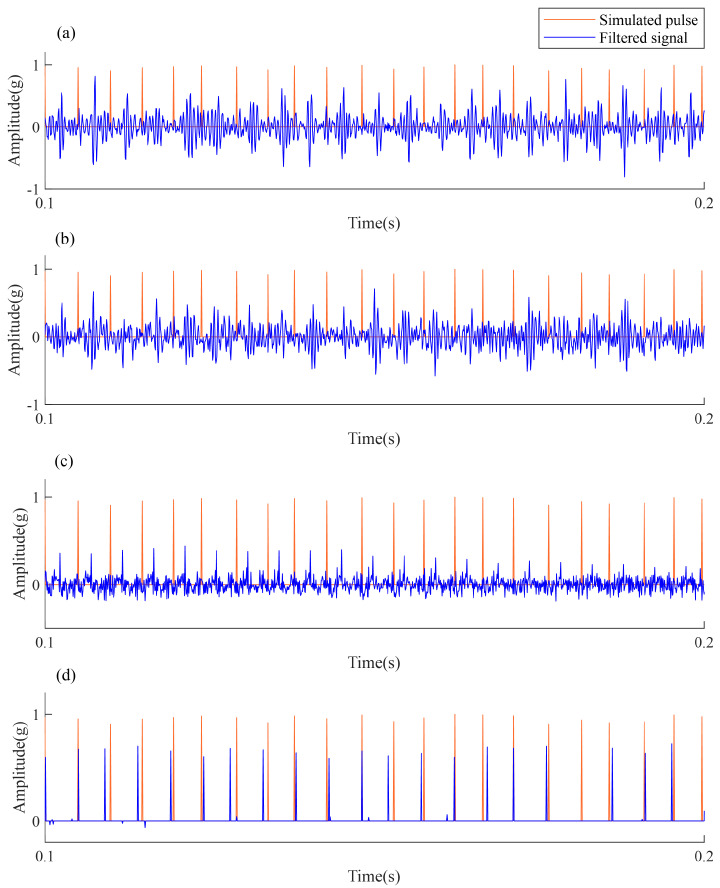
Localized time-domain plot of the filtered results. (**a**) Filtered signal of MCKD, (**b**) filtered signal of CYCBD, (**c**) filtered signal of BAD-MOMED, (**d**) filtered signal of the AMD-SUDN.

In order to further analyze the impulsiveness and periodicity of the pulse features extracted by each method, the time waveforms of the pulse features and the pulse sequences of the simulated signal are magnified and compared. The waveforms from 0.1 to 0.2 s are analyzed and shown in Figure 7. The orange line indicates the real pulse sequence, and the blue line indicates the deconvolution results of each method. Although MCKD, CYCBD and BAD-MOMED can recover the impulsiveness accurately, the recovered pulses do not match the real pulse sequence well. On the contrary, the proposed AMD-SUDN recovers the impulsive property of extracted sequence well, but also the periodicity structure of the extracted sequence is in line with the actual periodic fault information. Therefore, the proposed AMD-SUDN method achieves an excellent deconvolution performance by simultaneously retaining the waveform impulsiveness and periodicity cyclostationarity.

Finally, the computational efficiency of the four methods is determined, and the time costs are 0.83 s for MCKD, 0.98 s for CYCBD, 1.47 s for BAD-MOMED and 1.84 s for the AMD-SUDN, which indicates that the computational efficiency of the AMD-SUDN method is acceptable.

## 5. Experiment Verification

### 5.1. Case 1

**Figure 8:**
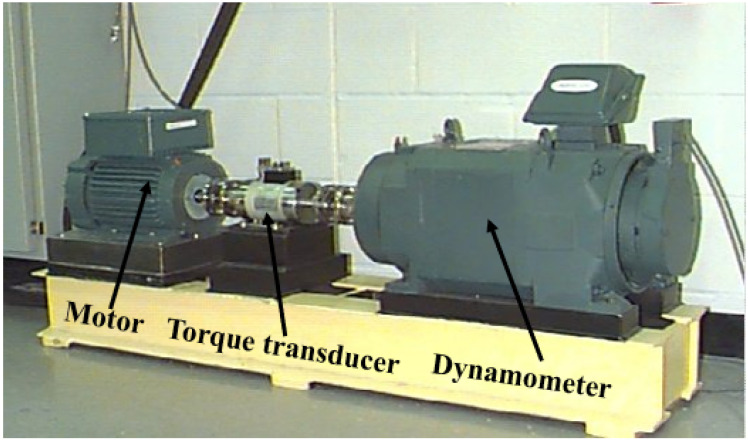
The test rig for bearing faults.

To further validate the superiority of the proposed AMD-SUDN in engineering applications, experimental bearing fault signals are analyzed. The bearing signals were downloaded from the Case Western Reserve University (CWRU) Bearing Data website. As shown in Figure 8, the test rig consisted of a motor, a torque transducer, and a dynamometer. An electro-discharge machine was used to induce a single point of failure in the test rolling bearing. The faulty bearing was mounted on the drive end of the motor. Accelerometers were placed on the drive and the fan ends of the motor housing.

**Figure 9:**
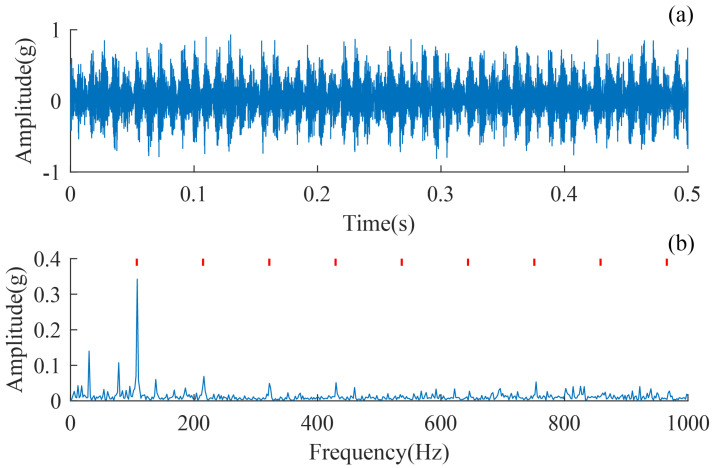
Raw signal of rolling bearing with an outer race defect. (**a**) Waveform of the signal, (**b**) envelope spectrum of the signal.

Outer ring failure is one of the common types of failure in rolling bearings. The vibration signals collected at the fan end were used to verify the proposed AMD-SUDN. The data number is X130-FE-time. The first 6000 data points were adopted as raw signals to test the proposed method. The raw signals are shown in Figure 9a. Periodic pulses excited by rolling bearing faults can be observed in the time-domain waveform, but these pulses are not sharp or significant due to transmission path influences. From the envelope spectrum in Figure 9b, only a first-order FCF can be observed, and no higher-order harmonics can be seen. This is not conducive to accurately judge the health of the rolling bearing.

**Figure 10:**
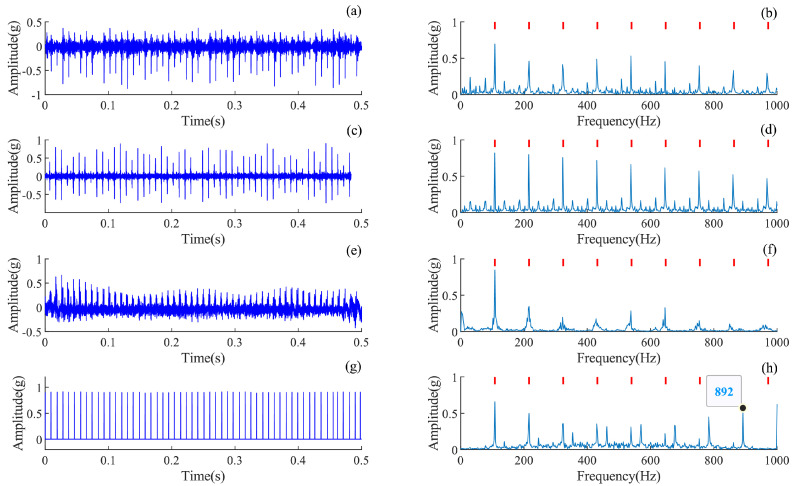
Deconvolution results of the rolling bearing with an outer race defect. (**a**) Filtered signal of MCKD and (**b**) its envelope spectrum, (**c**) filtered signal of CYCBD and (**d**) its envelope spectrum, (**e**) filtered signal of BAD-MOMED and (**f**) its envelope spectrum, (**g**) filtered signal of the AMD-SUDN and (**h**) its envelope spectrum.

The deconvolution results of all methods are shown in Figure 10. All comparative methods can enhance the impulsiveness of fault sources by mitigating the modulation influences of the transmission path. The proposed AMD-SUDN method recovers all impulses in the fault sources and the FCF orders are enhanced up to seven; more importantly, there is no noise in the time waveform and the periodicity among discrete pulses is significant. It is worth noting that the envelope spectrum of the comparison method may be superior to that of the proposed method because the downsampling operation of the MaxPooling layer reduces the frequency from 12 kHz to 1 kHz, so the frequency range of the faulty pulses extracted from the envelope spectrum will be reflected back at 1 kHz, which will result in some frequency mixing. However, MCKD, CYCBD and BAD-MOMED methods do not remove strong noise or interference components in the recovered impulsive sources; moreover, it is difficult to identify the periodicity pattern directly from the impulsive waveform. Therefore, it can be concluded that the proposed AMD-SUDN has a superior deconvolution performance and achieves a state-of-the-art accuracy.

### 5.2. Case 2

**Figure 11:**
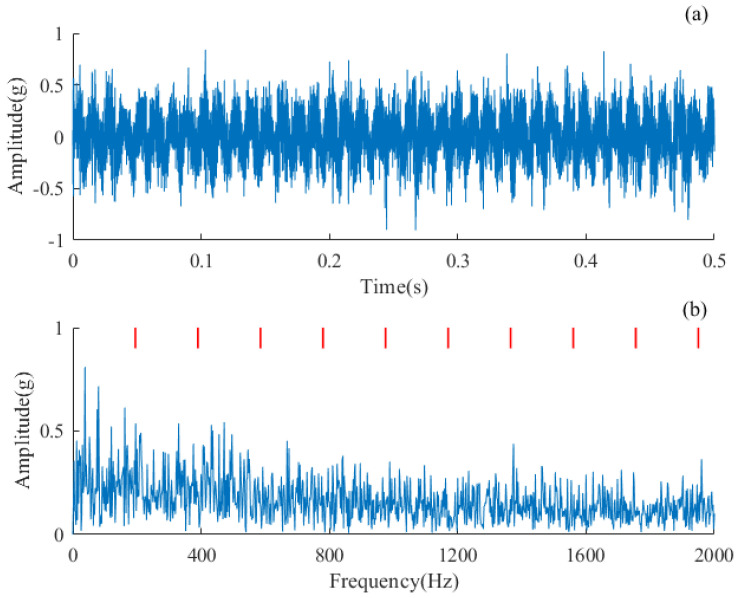
Raw signal of an inter-shaft bearing with an outer race defect. (**a**) Waveform of the signal, (**b**) envelope spectrum of the signal.

To further validate the feasibility of the proposed AMD-SUDN in the application of aero-engine bearing fault diagnosis, aero-engine inter-shaft bearing fault signals were analyzed. The aero-engine inter-shaft bearing fault signals were obtained from the Harbin Institute of Technology (HIT) [49], and in this subsection, a set of outer-ring fault signals are used to validate the proposed network, in which the low-pressure rotor rotates at a frequency of 50 Hz and the high-pressure rotor rotates at a frequency of 80 Hz, which is calculated to have an FCF of 195 Hz. A total of 12,500 data points are used as the raw signals to test the proposed method. The fault signal of the outer ring of the inter-shaft bearing is shown in Figure 11a. From the time-domain waveforms, it can be observed that the bearing fault features are overwhelmed by the harmonic components of the high-voltage rpm, and it is difficult to observe the fault feature waveforms therein. From the envelope spectrum in Figure 11b, only the first-order high-voltage rotor frequency can be clearly observed, there are also a large number of interference spectral lines, and the FCFs of the outer ring cannot be observed.

**Figure 12:**
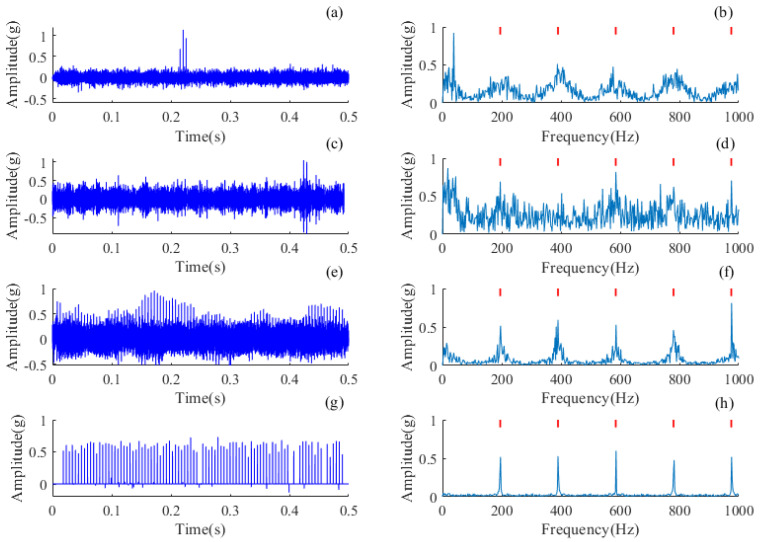
Deconvolution results of the inter-shaft bearing with an outer race defect. (**a**) Filtered signal of MCKD and (**b**) its envelope spectrum, (**c**) filtered signal of CYCBD and (**d**) its envelope spectrum, (**e**) filtered signal of BAD-MOMED and (**f**) its envelope spectrum, (**g**) filtered signal of AMD-SUDN and (**h**) its envelope spectrum.

The inverse convolution results for each method are shown in Figure 12. Since all the selected comparison methods require the input of a theoretical fault signature frequency, all the comparison methods are able to enhance the fault signature in the frequency domain. The proposed AMD-SUDN method recovers all the pulses in the fault source and enhances the FCF to the fifth order, and more importantly, there is no noise in the time waveform and the periodicity between the discrete pulses is significant. However, the MCKD, CYCBD and BAD-MOMED methods are affected by strong disturbances, retaining strong noise or interference components in the recovered pulse sources, and it is difficult to recognize periodic patterns directly from the pulse waveforms. Therefore, it can be concluded that the proposed AMD-SUDN has a superior deconvolution performance and achieves a superior accuracy.

## 6. Conclusions

In this article, an adaptive multi D-norm is firstly designed to simultaneously retain the waveform impulsiveness and periodicity cyclostationarity of bearing fault sources. A sparse unfolding deconvolution network is then established by unfolding the iterative steps of the convolutional sparse learning optimizer. By maximizing the adaptive multi D-norm to find desirable network parameters, an adaptive multi-D-norm-driven sparse unfolding deconvolution network (AMD-SUDN) is proposed for bearing fault diagnosis. A series of numerical simulations are implemented, and the comparative results verified that the proposed AMD-SUDN can satisfactorily retain the impulsiveness and cyclostationarity of fault sources. The feature mapping of every layer of the AMD-SUDN approximates the real fault sources, which provides an excellent strategy to design an interpretable network based on a physical model. More importantly, the adaptive multi D-norm objective function is free from strict periodic assumptions because MaxPooling provides a feasible way to adaptively decide impulse locations. Finally, its application to bearing fault diagnosis verifies that the AMD-SUDN achieves a higher deconvolution accuracy. All comparative results demonstrate that the AMD-SUDN method outperforms state-of-the-art deconvolution techniques.

Although the proposed AMD-SUDN can effectively deconvolve fault pulses, its envelope spectrum is easily affected by the downsampling of MaxPooling, and the parameters of MaxPooling need to be selected empirically. Therefore, we will continue to optimize the adaptive multi D-norm to remove the influence of MaxPooling and further consider a data noise reduction technique to highlight the fault features in rolling bearings so as to improve the network’s deconvolution effect.

## Figures and Tables

**Table 1:** Parameters of the simulation signal.

Fs (Hz)	FCF (Hz)	Amplitude (g)	SNR (dB)	ξ	$f_{d}$ (Hz)
12,000	210	1	−5	1256	2000

**Table 2:** Parameter selection for the AMD-SUDN.

*K*	*L*	Multi D-Norm	Times (s)
	100	6.65	1.80
	200	8.05	1.73
3	300	8.36	1.84
	400	8.59	1.84
	500	8.51	1.80
2		8.39	1.55
3		8.59	1.84
4	400	8.58	2.26
5		8.59	2.29
6		8.57	3.07

## Data Availability

Data are contained within the article.
